# Cortical spreading depolarisation-induced facial hyperalgesia, photophobia and hypomotility are ameliorated by sumatriptan and olcegepant

**DOI:** 10.1038/s41598-020-67948-w

**Published:** 2020-07-10

**Authors:** Chunhua Tang, Miyuki Unekawa, Satoshi Kitagawa, Tsubasa Takizawa, Yohei Kayama, Jin Nakahara, Mamoru Shibata

**Affiliations:** 1 Department of Neurology, Keio University School of Medicine, 35 Shinanomachi, Shinjuku-ku, Tokyo 160-8582 Japan; 2 Department of Neurology and Centre for Clinical Neuroscience, Daping Hospital, Third Military Medical University, 10 Changjiang Branch Road, Yuzhong District, Chongqing, 400042 China

**Keywords:** Migraine, Chronic pain

## Abstract

Cortical spreading depolarisation (CSD), the neural mechanism underlying migraine aura, may cause headache by sensitising the trigeminal system. Photophobia, the most bothersome accompanying symptom during migraine attacks, is more prevalent in migraine with aura than in migraine without aura. Whether CSD plays a role in developing photophobia remains unknown. Moreover, migraine-induced physical hypoactivity contributes to loss of productivity. We aimed to investigate the development of trigeminal sensitisation, photophobia and locomotive abnormality after KCl-induced CSD using 86 male C57BL/6 mice. Sham-operated mice were used as controls. We confirmed the presence of trigeminal sensitisation and photophobia at 24 h after CSD. CSD-subjected mice also exhibited significantly reduced locomotive activity in both light and dark zones. Hence, the CSD-induced hypomobility was likely to be independent of photophobia. The 5-HT_1B/1D_ agonist, sumatriptan, corrected all these CSD-induced abnormalities. Moreover, dose dependency was demonstrated in the ameliorating effect of the calcitonin gene-related peptide (CGRP) receptor antagonist, olcegepant, on these abnormalities. Sumatriptan and olcegepant improved mouse locomotion with therapeutic lags ranging from 20 to 30 min. Collectively, CSD caused trigeminal sensitisation, photophobia and hypomobility that persisted for at least 24 h by a mechanism involving the 5-HT_1B/1D_ and CGRP activity.

## Introduction

Cortical spreading depolarisation (CSD) is a wave of abrupt and sustained near-complete breakdown of transmembrane ion gradient and mass depolarisation concentrically spreading at ~ 1.5 to 9.5 mm/min in the grey matter of the brain^1^. In most cases, CSD suppresses the spontaneous activity of the brain tissue for several minutes. CSD was first reported in the rabbit brain by Leão^2^. Prior to this pioneering work, Lashley^3^ posited that the scotoma of migraine visual aura could be explained by a wave of intense excitation propagating at 3 mm/min across the visual cortex. In 1958, Milner^4^ reported a possible association between CSD and migraine visual aura. The development of CSD during migraine aura is supported by blood flow changes^5–9^ and complex direct current magnetoencephalographic shifts^10^. The most convincing evidence in humans for the association between CSD and migraine aura came from observed alterations in blood oxygenation level-dependent (BOLD) magnetic resonance imaging (MRI) compatible with the visual percept experienced during an episode of visual aura ^11^. Based on these studies, CSD is speculated as the pathophysiological mechanism underlying migraine aura. In addition, CSD has been shown to activate the trigeminal system^12–14^, implying that CSD may be responsible for migraine headache and migraine aura.

Photophobia is a well-known accompanying symptom of migraine^15^. During migraine attacks, photophobia can be improved by triptans (5-HT (serotonin)_1B/1D_ agonists) and calcitonin gene-related peptide (CGRP) receptor antagonists^16–18^. However, photophobia-associated symptoms are experienced by migraineurs not only during attacks but also in the interictal period, thus persistently impairing the quality of life^15^. Previous questionnaire-based studies demonstrated that patients affected by migraine with aura (MA) are more likely to exhibit these photophobia-associated symptoms than those affected by migraine without aura (MO) in both ictal and interictal phases^19,20^. Furthermore, in BOLD MRI studies and a sound-induced visual illusion test, MA patients showed greater excitability of the occipital cortex to visual stimuli compared with MO patients^19,21,22^. These findings raise the following two possibilities. Either CSD plays a role in forming a predisposition to photophobia or a hyperexcitable state in the occipital cortex is the primarily relevant pathophysiology in MA, which leads to the development of photophobia and CSD. However, this chicken-and-egg question is difficult to answer in clinical settings.

Migraine attacks render patients less mobile because migraineurs are aware that headache can be aggravated by physical activity. There are quantitative data showing that migraine reduces activity^23^. Hence, locomotive activity serves as an index to evaluate functionality of migraine patients.

In the present study, we aimed to investigate whether CSD can cause trigeminal sensitisation, photophobia and hypomotility in mice. We measured heat pain threshold temperature and mouse locomotion in a light/dark box equipped with an infrared tracking system. Furthermore, we examined the effects of intraperitoneal administration of sumatriptan and olcegepant on CSD-induced changes in these measures. In particular, we first succeeded in delineating the temporal profiles of the effects of these agents on CSD-induced mobility alterations.

## Results

### Experimental timelines, physiological parameters and CSD-related electrophysiological measures

Five different experimental groups were included in the present study. Sham-operated mice administered with the vehicle were designated as the Sham-Vehicle group. Vehicle-treated mice that underwent the CSD procedure were referred to as the CSD-Vehicle group. Those subjected to both CSD and pharmacological treatment were designated in accordance with the administered agent and dose: the CSD-Sumatriptan, CSD-Olcegepant 0.25 and CSD-Olcegepant 1.0 groups.

**Figure 1:**
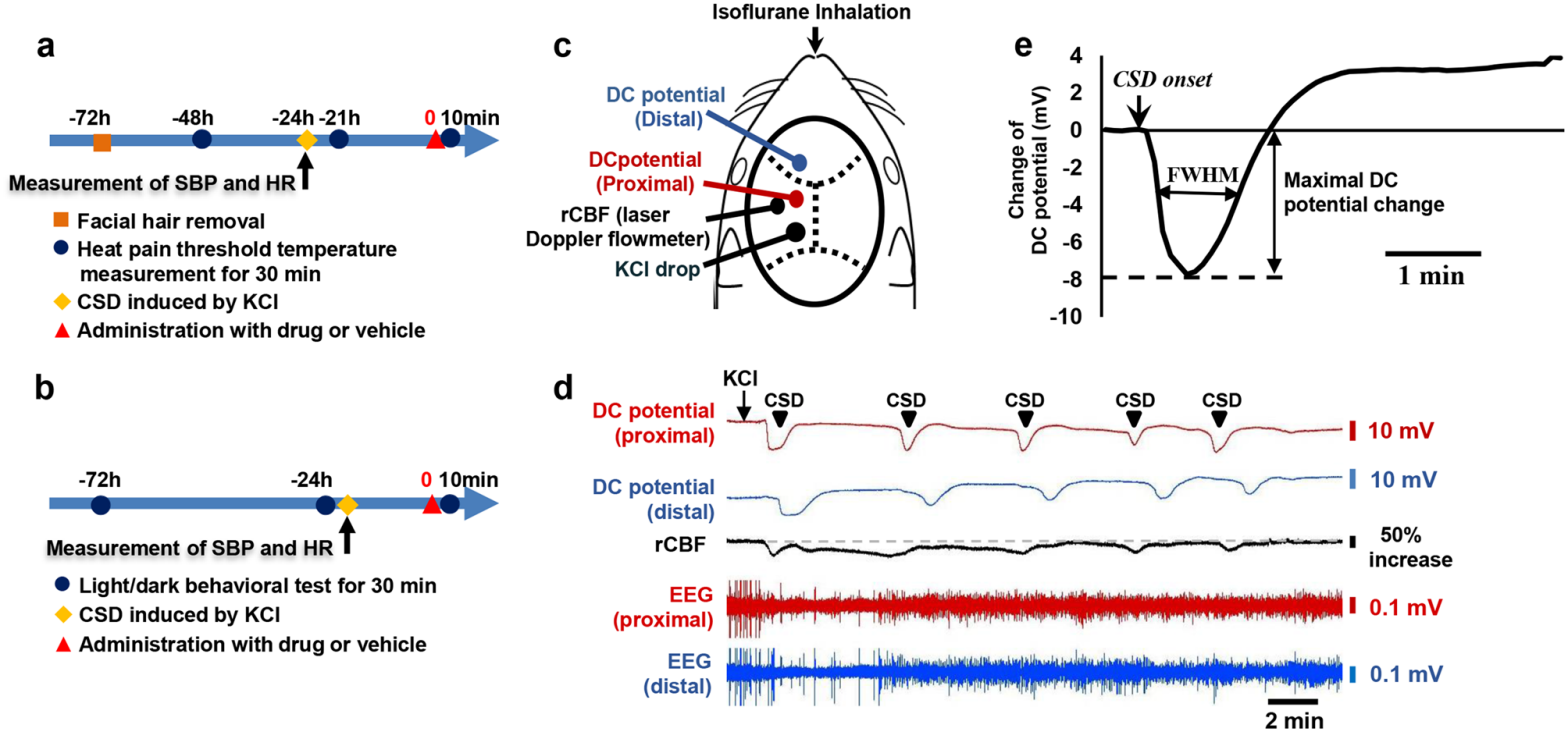
Experimental design, physiological parameters and cortical spreading depolarisation (CSD) induction. (**a**) Experimental timeline of facial heat pain threshold temperature measurement. (**b**) Experimental timeline of the light/dark box behavioural test. (**c**) Cartoon depicting the experimental setting for inducing CSD. rCBF: regional cerebral blood flow. (**d**) Representative recording of a CSD induction. (**e**) Electrophysiological parameters relevant to CSD are presented along with a cartoon depicting how the full width at the half-maximal time (FWHM) was measured. The 5-s temporal average of direct current (DC) potentials was recorded at the distal electrode. Time 0 was set at the onset of the DC potential decrease. The maximum change of the DC potential deflection and the FWHM were determined from the curves recorded at the distal electrode.

Experimental timelines for facial heat pain threshold temperature measurement and locomotive assessment are shown in Fig. 1a, b, respectively. Craniotomies for CSD induction and installation of experimental probes are shown in Fig. 1c. Intraoperative hemodynamic parameters (heart rate [bpm] and systolic blood pressure [mmHg]) and pre-operative body weight [g]) did not differ among the groups (Table 1). A representative recording of CSD induction is presented in Fig. 1d. As for the animals subjected to CSD induction, no significant between-group differences were found regarding CSD-related electrophysiological parameters (propagation velocity, full width at half maximum [FWHM] and maximal direct current [DC] potential change; Fig. 1e). In addition, baseline locomotive activity did not differ among the groups (Supplementary Table 1).

**Table 1:** Physiological and CSD-related electrophysiological parameters among experimental groups.

	Sham-Vehicle	CSD-Vehicle	CSD-Sumatriptan	CSD-Olcegepant 0.25	CSD-Olcegepant 1.0
**Basic physiological parameters of light/dark test**					
HR (bpm)	437 ± 25	472 ± 34	469 ± 30	466 ± 37	464 ± 21
SBP (mmHg)	73 ± 13	67 ± 13	65 ± 9	68 ± 9	74 ± 14
Body weight (g)	22.4 ± 0.8	25.8 ± 1.5	25.8 ± 1.2	24.7 ± 0.8	23.3 ± 0.4
**Electrophysiological parameters of light/dark test**					
Propagation velocity (mm/min)	N/A	4.4 ± 0.3	4.2 ± 0.2	4.3 ± 0.3	4.3 ± 0.2
FWHM (s)	N/A	47.5 ± 2.5	45.3 ± 3.3	45.7 ± 2.9	45.7 ± 2.9
Max. DC change (mV)	N/A	− 8.2 ± 0.9	− 7.8 ± 0.6	− 9.1 ± 1.0	− 9.6 ± 1.0
**Basic physiological parameters of heat pain test**					
HR (bpm)	444 ± 13	431 ± 10	430 ± 4	432 ± 15	444 ± 8
SBP (mmHg)	65 ± 3	67 ± 2	74 ± 4	56 ± 1	61 ± 3
Body weight (g)	22.1 ± 0.3	22.4 ± 0.2	22.5 ± 0.6	20.6 ± 0.2	22.1 ± 0.2
**Electrophysiological parameters of heat pain test**					
Propagation velocity (mm/min)	N/A	4.3 ± 0.2	4.3 ± 0.1	4.5 ± 0.2	4.2 ± 0.2
FWHM (s)	N/A	49.7 ± 3.2	48.1 ± 2.7	57.2 ± 5.2	47.4 ± 2
Max. DC change (mV)	N/A	− 6.5 ± 0.4	− 7.9 ± 1.2	− 6.5 ± 1.0	− 6.1 ± 1.0

### Temporal profiles of CSD-induced facial hyperalgesia

**Figure 2:**
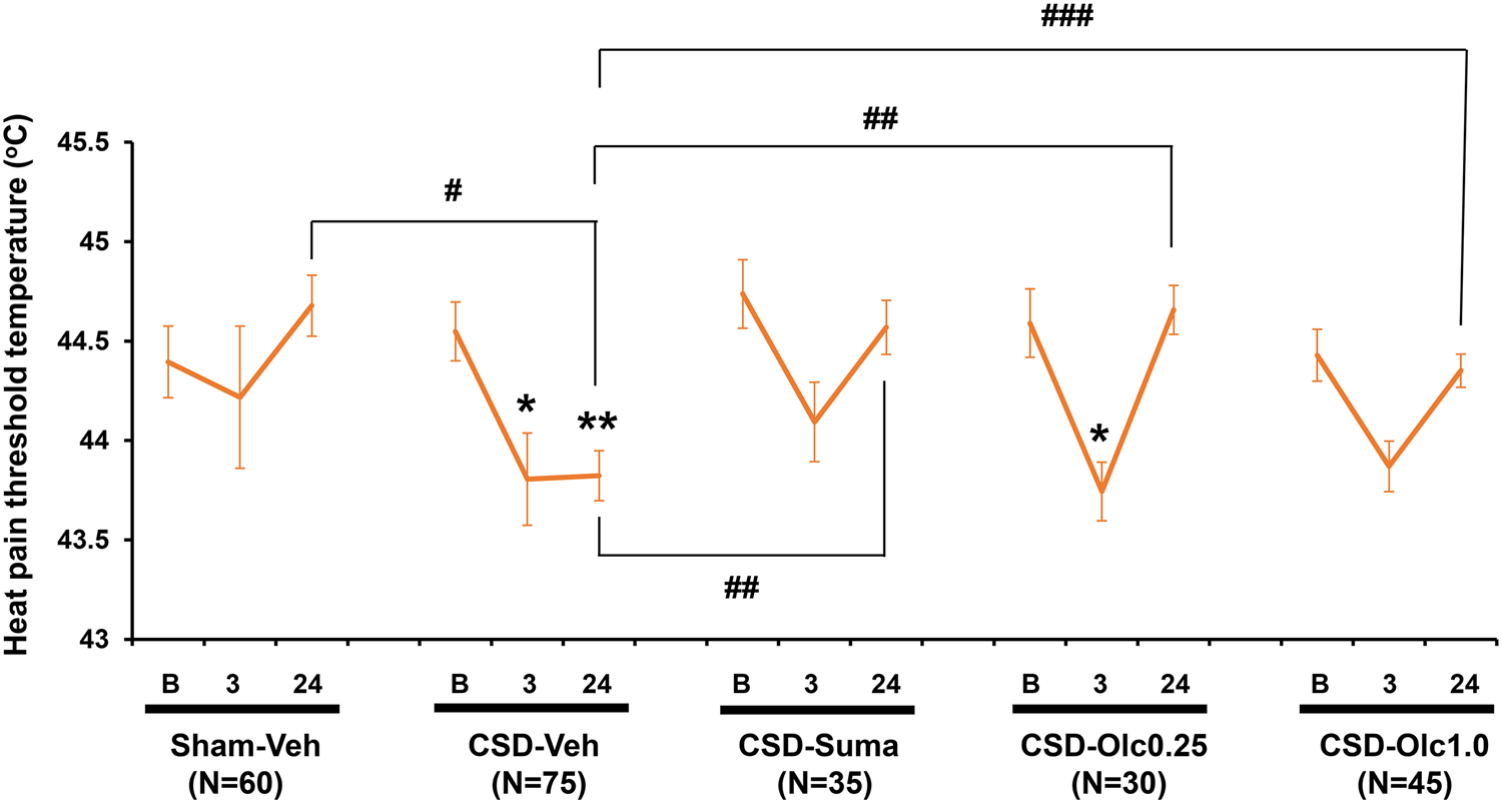
Temporal profiles of facial hyperalgesia. The ordinate indicates facial heat pain threshold temperature (°C). The two-way repeated measures analysis of variance was used to evaluate the effects of cortical spreading depolarisation (CSD) induction and pharmacological interventions on the parameters. Multiple comparisons were made by Bonferroni’s test (*P < 0.05, **P < 0.01 vs. First segment; ^#^*P* < 0.05, ^##^*P* < 0.01, ^###^*P* < 0.001 vs. CSD-Vehicle group at 24 h). *B* Baseline, *3* 3 h after CSD, *24* 24 h after CSD, *Sham-Veh* Sham-Vehicle group, *CSD-Veh* CSD-Vehicle group, *CSD-Suma* CSD-Sumatriptan group, *CSD-Olc0.25* CSD-Olcegepant 0.25 group, *CSD-Olc1.0* CSD-Olcegepant 1.0 group.

In the Sham-Vehicle group, no significant change was found in heat pain threshold temperature at 3 h and 24 h from the baseline value (Fig. 2). With regard to comparison between the Sham-Vehicle and CSD-Vehicle groups, the two-way analysis of variance (ANOVA) revealed a significant main effect of CSD induction (F_(1, 292)_ = 4.728, *P* = 0.0308) with a significant interaction between CSD induction and observation time (F_(2, 292)_ = 5.442, *P* = 0.0048; Fig. 2). At 24 h after CSD, heat pain threshold temperature was significantly lower in the CSD-Vehicle group than in the Sham-Vehicle group (mean difference: − 0.86 [95% confidence interval (CI) − 1.39 to − 0.32] °C, *P* = 0.0004, Bonferroni’s multiple comparison test; Fig. 2). Sumatriptan significantly altered the time course of the CSD-induced heat pain threshold temperature by inhibiting the CSD-induced reduction (F_(1, 245)_ = 6.332, *P* = 0.0125; Fig. 2). With regard to olcegepant, the two-way ANOVA did not detect an overall effect of olcegepant treatment on heat pain threshold temperature, but a significant difference was found at 24 h after CSD between the CSD-Vehicle and CSD-Olcegepant 1.0 groups (mean difference: − 0.53 [95% CI − 1.01 to − 0.05] °C, *P* = 0.0244, Bonferroni’s multiple comparison test; Fig. 2).

### CSD-induced reduction in total time spent in the light zone

Our preliminary assay revealed that normal male mice spent approximately 60% of the entire time in the dark zone under the lighting condition stated in the Methods section. Hence, our lighting condition was suitable for assessing photophobia.

**Figure 3:**
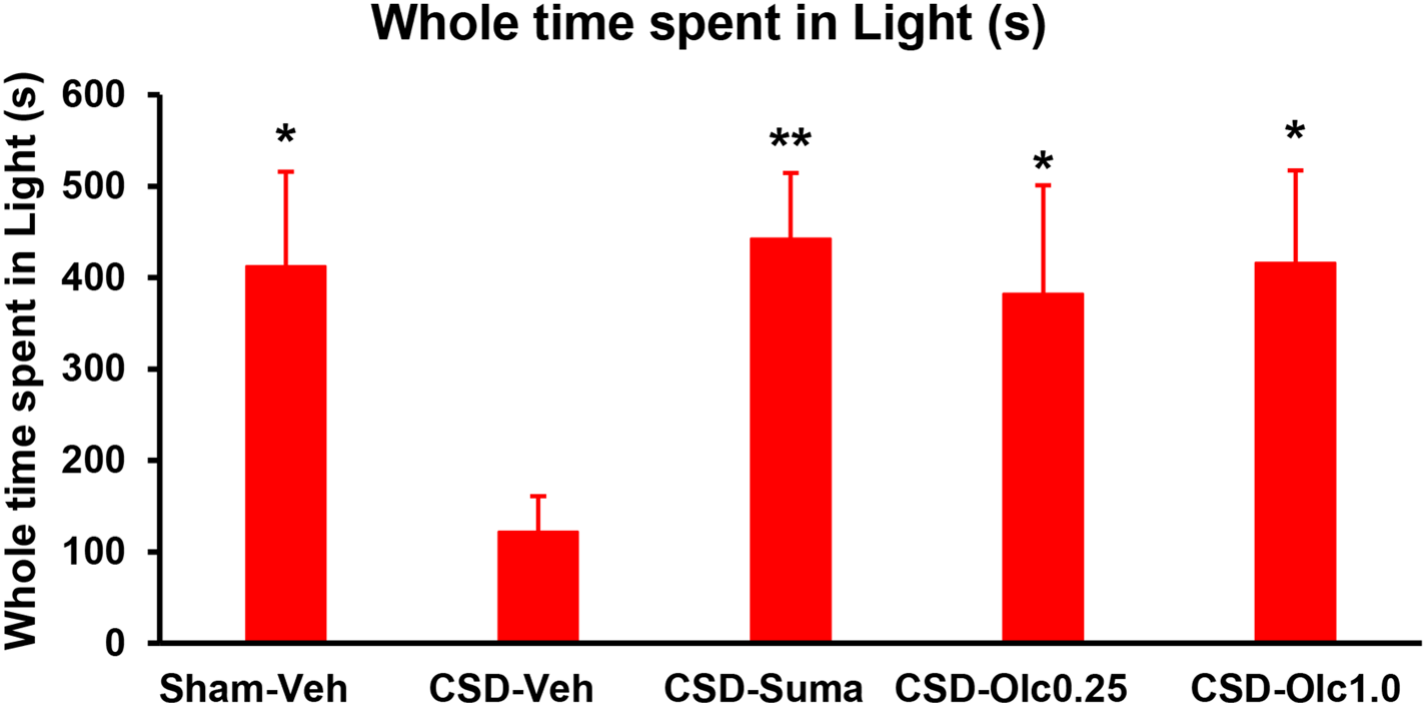
Total time spent in the light zone in each group. Statistical analysis was performed using the Kruskal–Wallis test, followed by Dunn’s multiple comparison test. *P < 0.05, **P < 0.01 versus CSD-Vehicle group. N = 8 in each group.

Compared with the Sham-Vehicle group, the CSD-Vehicle group spent significantly less time in the light zone (121.9 ± 38.9 s vs. 412.2 ± 103.8 s, *P* = 0.0021, Dunn’s multiple comparison test; Fig. 3). In the comparison among CSD-subjected mice, the total time spent in the light zone was significantly longer in sumatriptan-treated versus vehicle-treated mice (CSD-Sumatriptan group: 442.4 ± 71.9 s vs. CSD-Vehicle group: 121.9 ± 38.9 s, *P* = 0.0021, Dunn’s multiple comparison test; Fig. 3). A trend was observed that olcegepant exerted an ameliorating effect at 0.25 mg/kg (CSD-Olcegepant 0.25 group: 382.4 ± 118.6 s, *P* = 0.0664 vs. CSD-Vehicle group, Dunn’s multiple comparison test; Fig. 3). At a dose of 1 mg/kg, olcegepant significantly increased the total time spent in the light zone versus vehicle (CSD-Olcegepant 1.0 group: 416.1 ± 101.1 s, *P* = 0.0184, Dunn’s multiple comparison test; Fig. 3).

### CSD-induced changes in ambulatory time and ambulatory distance in the light zone

**Figure 4:**
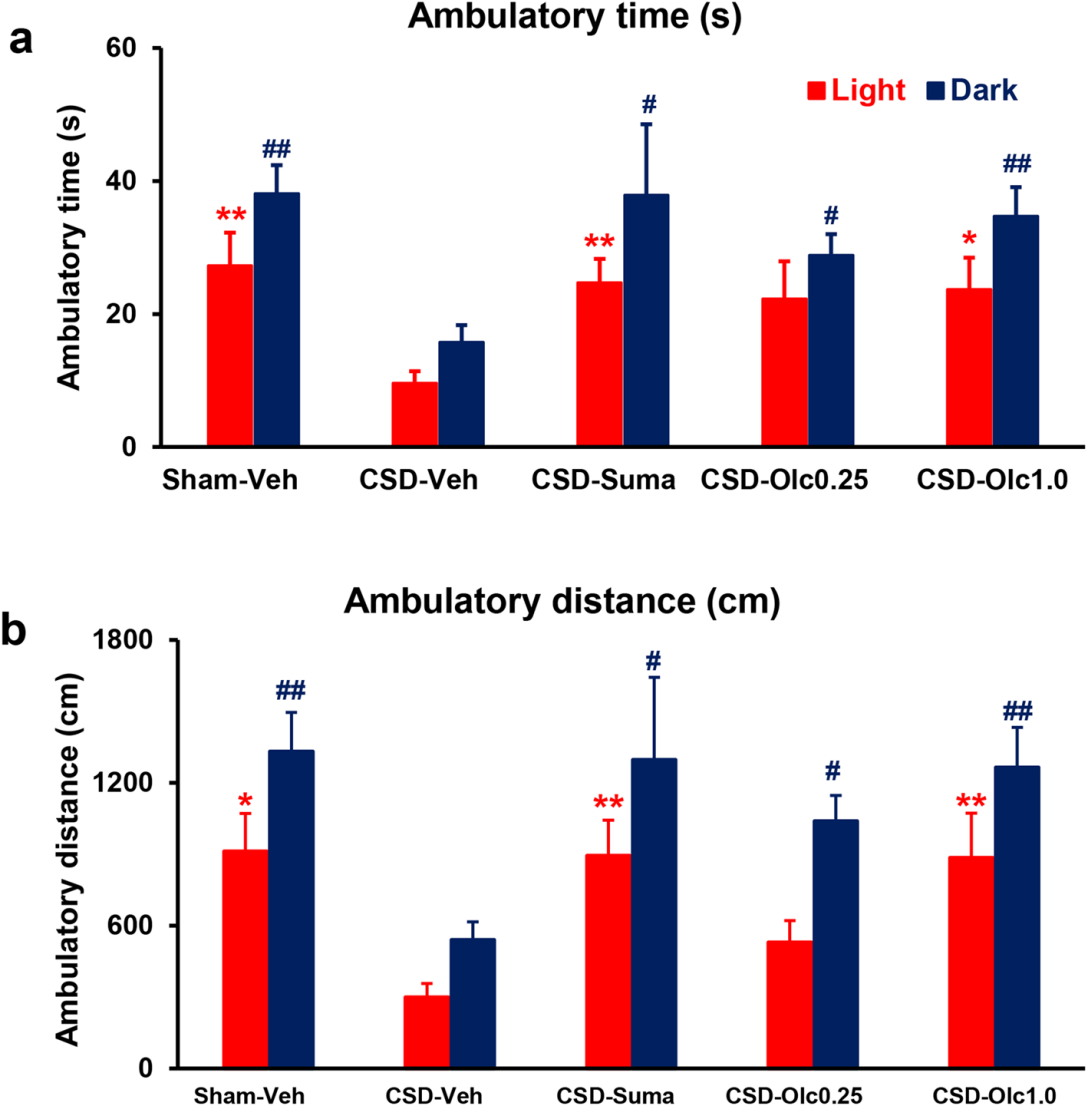
Ambulatory time and ambulatory distance in the light and dark zones. Red and blue bars represent the light and dark data, respectively. (**a**) The ordinate indicates the ambulatory time (s). (**b**) The ordinate indicates the ambulatory distance (cm). Statistical analysis was performed using the Kruskal–Wallis test, followed by Dunn’s multiple comparison test. *P < 0.05, **P < 0.01 versus CSD-Vehicle group. N = 8 in each group.

In the light zone, the CSD-Vehicle group exhibited a significantly shorter ambulatory time than the Sham-Vehicle group (9.6 ± 1.8 s vs. 27.3 ± 5.0 s, *P* = 0.0024, Dunn’s multiple comparison test; Fig. 4a). Sumatriptan and olcegepant (1.0 mg/kg) significantly improved the shortening of ambulatory time (24.7 ± 3.6 s in the CSD-Sumatriptan group, *P* = 0.0055 and 23.7 ± 4.8 s in the CSD-Olcegepant 1.0 group, *P* = 0.034, Dunn’s multiple comparison test; Fig. 4a). Similarly, the ambulatory distance travelled was shorter in the CSD-Vehicle group than in the Sham-Vehicle group (299 ± 59 cm vs. 911 ± 161 cm, *P* = 0.0118, Dunn’s multiple comparison test; Fig. 4b). Sumatriptan and olcegepant (1.0 mg/kg) reversed the CSD-induced reduction in ambulatory distance (893 ± 150 cm in the CSD-Sumatriptan group, *P* = 0.0026 and 885 ± 188 cm in the CSD-Olcegepant 1.0 group, *P* = 0.0085, Dunn’s multiple comparison test; Fig. 4b).

### CSD-induced changes in ambulatory time and ambulatory distance in the dark zone

In the dark zone, the CSD-Vehicle group was significantly less ambulatory compared with the Sham-Vehicle group with regard to both time (15.8 ± 2.6 s vs. 38.1 ± 4.3 s, *P* = 0.0014, Dunn’s multiple comparison test; Fig. 4a) and distance (539 ± 76 cm vs. 1,331 ± 164 cm, *P* = 0.0023, Dunn’s multiple comparison test; Fig. 4b). All pharmacological interventions significantly prevented the CSD-induced reduction in ambulatory time (37.9 ± 10.7 s in the CSD-Sumatriptan group, *P* = 0.0292; 28.8 ± 3.2 s in the CSD-Olcegepant 0.25 group, *P* = 0.0396; and 34.7 ± 4.4 s in the CSD-Olcegepant 1.0 group, *P* = 0.005, Dunn’s multiple comparison test; Fig. 4a). In addition, all pharmacological interventions significantly ameliorated the CSD-induced decrease in distance travelled (1,296 ± 346 cm in the CSD-Sumatriptan group, *P* = 0.027; 1,038 ± 108 cm in the CSD-Olcegepant 0.25 group, *P* = 0.0426; and 1,264 ± 169 cm in the CSD-Olcegepant 1.0 group, *P* = 0.0034, Dunn’s multiple comparison test; Fig. 4b).

### Comparison of CSD-induced changes in ambulatory time proportion between the light and dark zones

**Figure 5:**
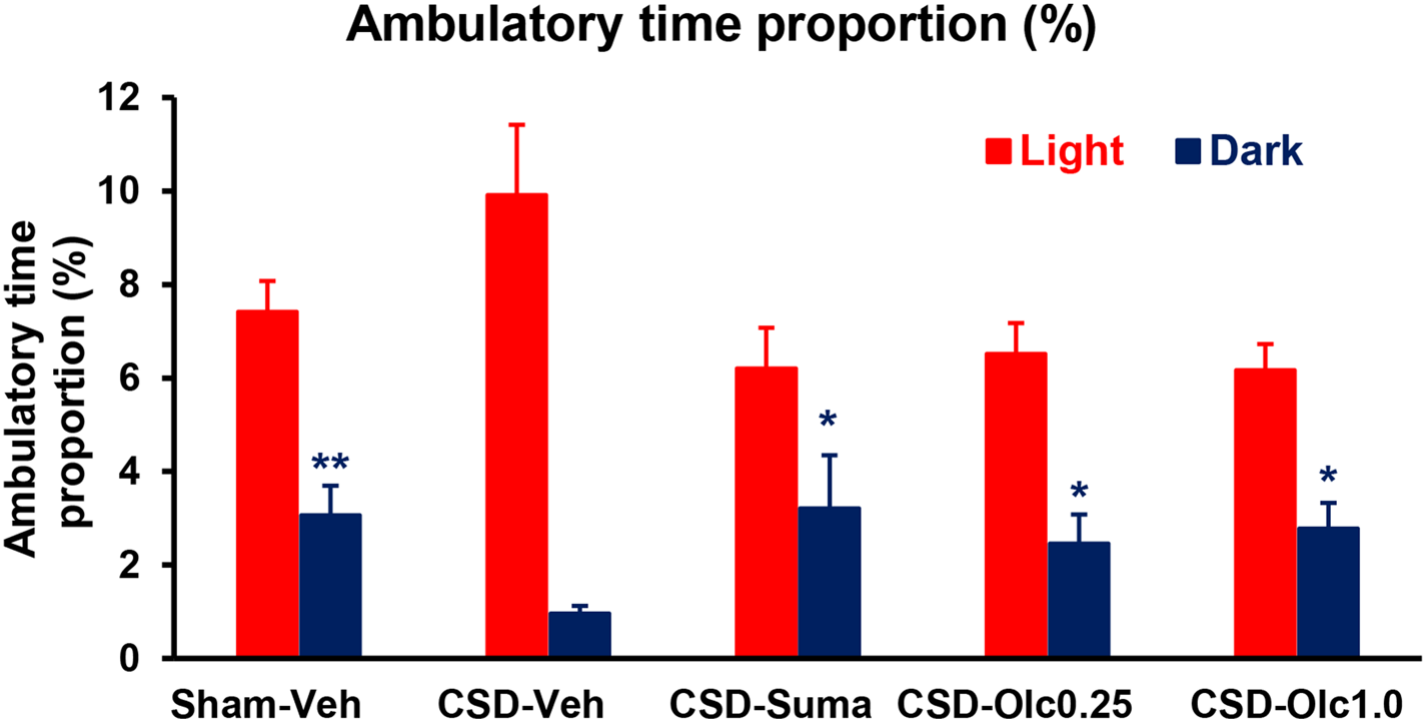
Comparison of cortical spreading depolarisation (CSD)-induced changes in ambulatory time proportion between the light and dark zones. Red and blue bars represent the light and dark data, respectively. The ordinate indicates the ambulatory time proportion (%). Statistical analysis was performed using the Kruskal–Wallis test, followed by Dunn’s multiple comparison test. *P < 0.05, **P < 0.01 versus CSD-Vehicle group. N = 8 in each group.

Ambulatory time proportion of total time spent in the light zone did not differ among the groups, although the CSD-Vehicle group exhibited an increasing trend compared with the Sham-Vehicle group (Fig. 5, red bars). In the dark zone, the CSD-Vehicle group exhibited a significant decrease compared with the Sham-Vehicle group (1.0 ± 0.2% vs. 3.1 ± 0.6%, *P* = 0.0029, Dunn’s multiple comparison test; Fig. 5, blue bars). This CSD-induced reduction in ambulatory time proportion was reversed by sumatriptan and olcegepant at 1.0 mg/kg (2.9 ± 0.8% in the CSD-Sumatriptan group and 2.8 ± 0.5% in the CSD-Olcegepant 1.0 group, *P* = 0.0156, Dunn’s multiple comparison test; Fig. 5, blue bar 4).

### Temporal profiles of the ambulatory time in the light zone

**Figure 6:**
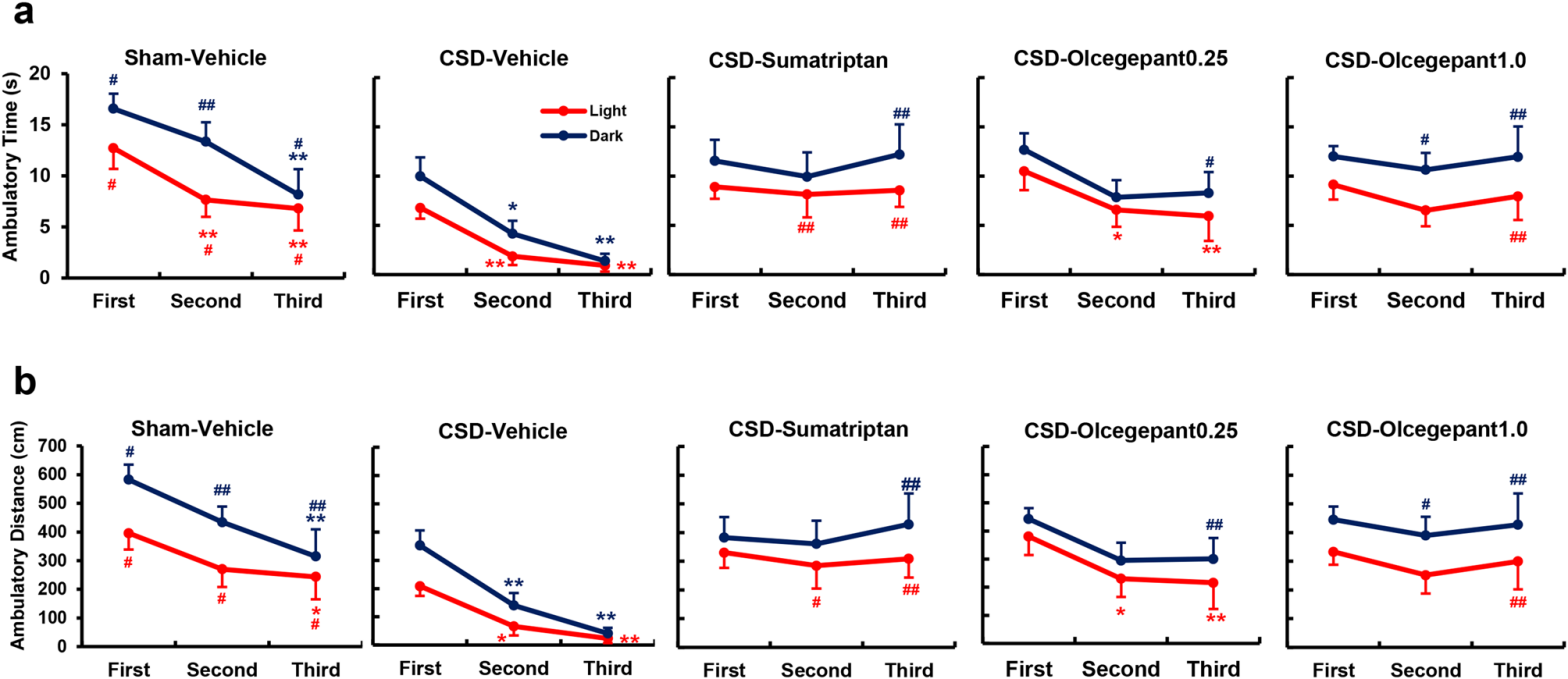
Temporal profiles of ambulatory time and ambulatory distance in the light and dark zones. Red and blue lines represent the light and dark data, respectively. (**a**) Time courses of the ambulatory time (s) are shown for each group. (**b**) Time courses of the ambulatory distance (cm) are shown for each group. The abscissa indicates 10-min-long-time segments (first, second and third). The two-way repeated-measures analysis of variance was used to evaluate the effects of cortical spreading depolarisation (CSD) induction and pharmacological interventions on the parameters. Multiple comparisons were conducted using Bonferroni’s test (*P < 0.05, **P < 0.01 vs. First segment; ^#^*P* < 0.05, ^##^*P* < 0.01 vs. CSD-Vehicle group in the same time segment). N = 8 in each group.

With regard to the comparison between the Sham-Vehicle and CSD-Vehicle groups, the two-way ANOVA revealed significant main effects of observation time (F_(2,28)_ = 17.26, *P* < 0.0001) and CSD induction (F_(1,14)_ = 11.15, *P* = 0.0049) on ambulatory time, whereas no significant interaction between them was observed (Fig. 6a). Sumatriptan exerted a significant main effect on ambulatory time (F_(1,14)_ = 12.53, *P* = 0.0033), but no significant interaction was noted between sumatriptan treatment and observation time (Fig. 6a). Significant interaction was not found between these factors (*P* = 0.2231). On the other hand, olcegepant showed no significant main effect on ambulatory time, although a significant difference was noted between the CSD-Vehicle and CSD-Olcegepant 1.0 groups only in the third segment (mean difference − 7.03 [95% CI − 12.56 to − 1.49] s, *P* = 0.0098; Fig. 6a).

### Temporal profiles of the ambulatory distance in the light zone

With regard to the comparison between the Sham-Vehicle and CSD-Vehicle groups, the two-way ANOVA detected significant main effects of observation time (F_(2, 28)_ = 10.56, *P* = 0.0004) and CSD induction (F_(1, 14)_ = 12.86, *P* = 0.003) on ambulatory distance in the light zone, but no significant interaction was found between these factors (Fig. 6b). Significant ameliorating effects of sumatriptan (F_(1, 14)_ = 12.4, *P* = 0.0034) and olcegepant (F_(2, 21)_ = 3.908, *P* = 0.00361) were noted on ambulatory distance in the light zone (Fig. 6b). No significant interaction was found between observation time and either of these drugs.

### Temporal profiles of the ambulatory time in the dark zone

Comparing the Sham-Vehicle and CSD-Vehicle groups, the two-way ANOVA found significant main effects of observation time (F_(2, 28)_ = 15.21, *P* < 0.0001) and CSD induction (F_(1, 14)_ = 20.12, *P* = 0.0005) on ambulatory time in the dark zone, whereas no significant interaction between them was detected (Fig. 6a). The CSD-induced reduction in ambulatory time in the dark zone was improved by both sumatriptan and olcegepant. Sumatriptan exerted a significant main effect (F_(1, 14)_ = 6.445, *P* = 0.0236) with a significant interaction with observation time (F_(2, 28)_ = 4.907, *P* = 0.0149; Fig. 6a). A significant main effect of olcegepant was found (F_(2, 21)_ = 7.942, *P* = 0.0027). No significant interaction was noted between observation time and olcegepant treatment (Fig. 6a).

### Temporal profiles of the ambulatory distance in the dark zone

With regard to the comparison between the Sham-Vehicle and CSD-Vehicle groups, the two-way ANOVA detected significant main effects of observation time (F_(2, 28)_ = 18.84, *P* = 0.0001) and CSD induction (F_(1, 14)_ = 19.68, *P* = 0.006) on ambulatory distance in the dark zone, whereas no significant interaction was found between these factors (Fig. 6b). The CSD-induced reduction in ambulatory distance in the dark zone was ameliorated by both sumatriptan and olcegepant. Sumatriptan exerted a significant main effect (F_(1, 14)_ = 6.833, *P* = 0.0204) with a significant interaction with observation time (F_(2, 28)_ = 7.567, *P* = 0.0024; Fig. 6b). A significant main effect of olcegepant on ambulatory distance was detected (F_(2, 21)_ = 8.969, *P* = 0.0015), but no interaction was observed with observation time (Fig. 6b).

### Comparison of the average ambulatory speed among experimental groups

The average ambulatory speed was measured in each mouse to examine whether CSD induction or pharmacological interventions caused motor impairments. Significant between-group differences were not observed in average ambulatory speed (Sham-Vehicle group: 52.6 ± 0.7 cm/s, CSD-Vehicle group: 54.0 ± 1.1 cm/s, CSD-Sumatriptan group: 52.4 ± 0.9 cm/s, CSD-Olcegepant 0.25 group: 53.3 ± 1.0 cm/s and CSD-Olcegepant 1.0 group: 57.8 ± 0.8 cm/s, *P* = 0.8224, Kruskal–Wallis test).

## Discussion

Our data on facial heat pain threshold temperature revealed the presence of facial hyperalgesia at 24 h after CSD induction. In addition, our locomotive analysis demonstrated that light aversion was present 24 h after CSD induction, as evidenced by a significantly reduced total time spent in the light zone. Moreover, CSD-affected mice presented significantly decreased values for ambulatory time and distance in both light and dark zones in similar profiles. Such reductions in ambulatory time and ambulatory distance could not be explained by motor impairment, because average ambulatory speed was not altered by CSD. The proportions of ambulatory time per total time spent significantly decreased in the dark zone, but tended to increase in the light zone. The former finding is reminiscent of the typical ictal behaviour pattern of migraineurs who prefer resting in a dark environment. The latter can be interpreted as an attempt of CSD-subjected mice to promptly evade from the aversive environment (light zone) to the more comfortable one (dark zone). In this sense, this observation seems to lend further support to the tenet that CSD is causative of photophobia. From a therapeutic perspective, both sumatriptan and olcegepant were effective in ameliorating facial hyperalgesia, photophobic behaviour and locomotive hypoactivity in the light and dark zones. Moreover, we demonstrated dose-dependent effects of olcegepant on these abnormalities.

CSD has been demonstrated to sensitise the trigeminal system^12–14,24^, although this has yet to be validated in humans. Facial hyperalgesia is a representative manifestation of trigeminal sensitisation^25,26^. We previously demonstrated that facial hyperalgesia was induced in an inflammatory soup-based headache model^27^. A salient finding in the present study is that CSD-induced trigeminal sensitisation persists for at least 24 h. CSD-subjected mice were inferred to be still in a condition similar to the ictal state of migraine at this timepoint. This tenet is reinforced by the fact that the facial hyperalgesia was reversed by acute anti-migraine drugs, sumatriptan and olcegepant.

Patients affected by migraine regard photophobia as the most bothersome accompanying symptom^15^. In contemporary society, people are prone to be exposed to a variety of light sources. For many people, working in front of a visual display terminal and using a smartphone on multiple occasions on a daily basis are inevitable. This holds true especially for young individuals, among whom migraine causes the highest disability rates^28,29^. Hence, proper management of ictal photophobic symptoms should decrease the migraine-induced loss of productivity by improving the functionality in patients with migraine^30,31^. The present study provides therapeutically important information that sumatriptan and olcegepant are efficacious in reversing CSD-induced photophobic behaviour.

Along with photophobia, reduced activity seems to affect a migraine-associated decrease in functionality. A quantitative study using ambulatory accelerometry reported that migraine reduces overall body motility in a manner dependent on attack severity^23^. The light/dark box test is traditionally used mainly to assess anxiety, activity and exploration^32^. With regard to the applicability of this experimental system to migraine research, Recober et al.^33^ succeeded in characterising the temporal profile of CGRP-induced behavioural pattern and light aversion. In the present study, we demonstrated that hypomotility existed in both light and dark zones 24 h after CSD, thus indicating that hypomotility is independent of photophobia. Of particular relevance, our study delineated the trajectories of the behavioural response to sumatriptan and olcegepant treatments. Our data showed that sumatriptan and olcegepant (1.0 mg/kg) exerted significant efficacy only in the second or third segments (20–40 min after treatment induction). Hence, these drugs seem to have therapeutic lags ranging from 20 to 30 min when administered intraperitoneally.

Collectively, sumatriptan and olcegepant were effective in ameliorating CSD-induced facial hyperalgesia, photophobia and hypomotility. A dose of 0.6 mg/kg sumatriptan used in our experiment has been shown to be effective in alleviating nitroglycerin-induced thermal and mechanical hyperalgesia^34^ and CGRP-induced photophobia^35^ in mice. Compared with sumatriptan, olcegepant has been used much less in rodent studies. Nevertheless, the efficacy of olcegepant against our measures seems to be specific, because its dose dependency was confirmed.

With regard to the efficacy of both drugs in CSD-induced facial hyperalgesia and hypomotility, interpreting that mouse locomotion was improved as a result of pain relief seems reasonable. What is the mechanism whereby sumatriptan and olcegepant alleviate CSD-induced photophobic behaviour? Noseda et al.^15^ postulated that the convergence of photic signals from intrinsically photosensitive retinal ganglion cells onto the trigeminovascular thalamocortical pathway plays an important role in the intensification of headache by light. By using single-unit recording and neural tract tracing in the rat, they identified dura-sensitive neurons in the posterior thalamus whose activity was modulated also by light and whose axons projected extensively to the somatosensory, visual and associative cortices^36^. Hence, sumatriptan and olcegepant are deduced to act somewhere along this neural pathway in our experimental model. Intriguingly, 5-HT_1B_ and 5-HT_1D_ receptors are enriched in the occipital cortex^37^. Moreover, recent evidence shows that extensive localisation of CGRP and its receptor exists in the central nervous system^38,39^. These findings raise the possibility that sumatriptan and olcegepant exerted their efficacy by a central mechanism. On the other hand, the ameliorating effect could possibly be exerted by a peripheral mechanism. Triptans is generally accepted to exert their anti-migraine efficacy by acting on 5-HT_1D_ receptors located at trigeminal terminals^40,41^. The localization of 5-HT_1D_ receptors on CGRP-positive trigeminal ganglion neurons has been demonstrated in humans^42^, and 5-HT_1D_ receptor blockade has been shown to block CGRP release from trigeminal terminals^43,44^. In addition, recent experimental data have highlighted the action of CGRP on a subset of trigeminal fibres innervating the dura^45,46^. The ability of sumatriptan and olcegepant to suppress pain signals at the nociceptor level may attenuate photophobia, as well as headache, because of decreased nociceptive input to the thalamus. Hence, determining the sites of action of these drugs in our experiments is difficult. Although sumatriptan does not readily penetrate the blood–brain barrier^47,48^, the invasive nature of our surgical procedure might have compromised its integrity. In this regard, using monoclonal antibodies targeting CGRP or its receptor^45,46,49^ and less invasive surgical techniques for inducing CSD^50^ may provide a clearer answer to this problem. The use of monoclonal antibodies may be beneficial in terms of target specificity as well, especially because we were able to obtain robust effects of olcegepant at the higher dose. Our experimental system is useful for assessing therapeutic efficacy with regard to trigeminal sensitisation, light aversion and hypoactivity in murine migraine models.

The present study has several limitations. First, since the assessment of locomotion in the light and dark was performed only at 24 h after CSD induction, how photophobia and hypomotility develop temporally after CSD remains unclear. The invasive nature of the CSD-related surgery and potential residual effects of the anaesthesia rendered the accurate evaluation of the mouse motility at earlier timepoints difficult. We utilised KCl solution to evoke CSD, because this is the most reliable method for inducing CSD in our experience. In this regard, the possibility that KCl per se served as noxious stimulation to the surrounding trigeminal terminals cannot be completely ruled out. Second, we used only male mice in the present study, because CSD biology has been shown to be affected by the oestrous cycle^51,52^ and sex steroid hormones^53^. However, this might be a weakness, because migraine is approximately three times more common in females than in males^54,55^.

In summary, the present study provided quantitative evidence that trigeminal sensitisation, photophobic behaviour and reduced locomotion were present at 24 h after CSD induction. Moreover, we demonstrated that sumatriptan and olcegepant were efficacious in reversing such CSD-induced abnormalities with detailed temporal profiles of their efficacy for hypomobility. From a clinical viewpoint, the present study supports the view that CSD is causative of headache (trigeminal sensitization), photophobia and hypomotility, all of which impair quality of life in migraine patients. As for the chicken-and-egg question on the relationship between migraine aura and photophobia stated above, our findings may explain why patients with MA are more likely to exhibit photophobic symptoms than those with MO.

## Methods

### Animals

The present study was approved by the Laboratory Animal Care and Use Committee of Keio University (No. 14084). All experimental procedures were performed in accordance with the university's protocols and EC Directive 86/609/EEC for animal experiments. Male C57BL/6 mice aged 8–10 weeks were purchased from CLEA Japan Inc. (Fujinomiya, Japan). A total of 86 mice were examined. They were housed in an ambient specific-pathogen-free condition with a 12-h light/dark cycle; free access to food and water was allowed.

### Drug administration

Sumatriptan succinate (Tokyo Chemical Industry Co., Ltd., Tokyo, Japan), dissolved in normal saline, was intraperitoneally administered at a dose of 0.6 mg/kg through a 26-gauge needle. The amount of diluent was 10 µL/g. Olcegepant (MedChemExpress, Mammoth Junction, NJ), initially dissolved in dimethylsufloxide at 0.025 mg/µL, was 250-fold diluted in normal saline. Intraperitoneal injections at a dose of either 1.0 mg/kg or 0.25 mg/kg were performed using a 26-gauge needle. Normal saline without dimethylsufloxide was used as the vehicle. Our preliminary data showed that mouse locomotion was affected by an intraperitoneal injection with normal saline, particularly in the 60-min post-injection period. Hence, sham-operated mice with an intraperitoneal injection of normal saline were used as controls in locomotion assays.

### CSD induction

Under isoflurane anaesthesia (1–2%), the mouse head was fixed in a stereotaxic apparatus. Systolic blood pressure and heart rate were monitored at the tail artery with a non-invasive blood pressure monitor (MK-2000ST; Muromachi Kikai Co., Ltd., Tokyo, Japan). Body temperature was maintained at approximately 37 °C using a heating-pad and thermocontroller (BWT-100; Bioresearch Center Co., Ltd., Nagoya, Japan).

The detailed procedures of the electrophysiological recording were described previously^56^. Schematic representations for surgery are presented in Fig. 1. Briefly, three holes were drilled in the skull over the left hemisphere. A posterior hole, approximately 1 mm in diameter centred at the coordinates of 5 mm posterior and 2 mm lateral to the bregma, was made for the KCl stimulation. The parietal hole (2 mm lateral and 2 mm caudal to the bregma) and the frontal hole (2 mm lateral and 2 mm rostral to the bregma) were made for the placement of the recording electrodes. The diameters of these holes were less than 1 mm. Two Ag⁄AgCl DC electrodes (tip diameter = 200 μm, EEG-5002Ag; Bioresearch Center Co., Ltd.) were placed on the dura at the parietal (proximal) and frontal (distal) holes, respectively, and fixed with dental cement. A DC potential was applied at 1–100 Hz and digitised at 1 kHz with a differential head stage and differential extracellular amplifier (Model 4,002 and EX1; Dagan Co., Minneapolis, MN, USA). In the area close to the parietal hole (4 mm lateral and 2 mm posterior to the bregma), the probe (BF52; Advance Co., Ltd., Tokyo, Japan) of a laser Doppler flowmeter (LDF; ALF 21, Advance Co., Ltd.) was installed on the intact skull to monitor the regional cerebral blood flow (rCBF). Continuous recordings of the DC potential and rCBF were stored on a multi-channel recorder (PowerLab 8/30; ADInstruments, Ltd., Sydney, Australia.), and LabChart software (ADInstruments, Ltd.) was used for off-line analysis as reported previously. After confirmation that all parameters had been stable for at least 10 min, CSD was induced by chemical stimulation with KCl solution (1.0 mol/L, 5 μL) onto the dura. The appearance of CSD was confirmed by the demonstration of a distinct DC potential deflection, typical fluctuation of rCBF, propagation to the distal portion and the suppression of electroencephalography (Fig. 1d). The CSD induction was performed five times after washing the cortical surface with normal saline to prevent residual KCl solution from acting on the nociceptors in the surrounding tissue. After the surgery, all electrodes were removed, and the craniotomies were closed off with dental cement. In sham-operated mice, all these surgical procedures were carried out except for the chemical stimulation with KCl solution and DC electrodes insertion. LDF was stalled on the skull to confirm no occurrence of CSD.

CSD-related electrophysiological parameters (propagation velocity, DC potential decrease and FWHM) were examined. CSD propagation velocity was calculated from the latency and distance between the proximal and distal electrodes. The maximum decrease in DC potential and FWHM were determined from the curves recorded at the distal electrode.

### Facial heat pain threshold temperature measurement

The detailed protocol for heat pain threshold temperature was described elsewhere^27^. Briefly, after acclimation to an experimental apparatus that restricted body mobility, save for head movement and facial hair removal, a pair of Peltier module bars with surface temperature regulated between 36 and 56 °C was applied to the face bilaterally. The bar surface temperature was gradually increased from 36 °C by 1 °C/4 s until face withdrawal. Mouse behaviours were monitored using a video recorder (Panasonic, Kadoma, Japan). The video was analysed by an examiner blind to the identity of the animals. The lowest temperature at which a mouse turned the head away from the bars was recorded as the heat pain threshold temperature. In each session, measurement of the threshold temperature was repeated five times. The measurement was carried out before surgery, and 3 and 24 h after surgery. At 24 h after surgery, the timing of measurement was between 10 and 30 min after administration with a drug or vehicle.

### Mouse locomotion analysis in the light and dark zones

Mouse behaviours were continuously monitored in an open field chamber (27 cm wide × 27 cm deep × 20.3 cm high) with three sets of 16-beam infrared arrays (two sets of perpendicular beams crossed at a height of 1.0 cm to detect mouse location and locomotion, and the third beam crossed the width of the chamber at a height of 7.3 cm to detect vertical activity; Med Associates, Fairfax, VT). The testing chamber was placed inside a windowless cabinet. The testing field was equally compartmented in light and dark zones by a dark insert (Med Associates). Mice could move freely between the two zones through an orifice (5.2 cm × 6.8 cm) in the dark insert. The light intensity measured at a height of 2 cm in the light zone was 540 lx, whereas the intensities measured in the dark zone were 380 lx immediately inside the orifice, 20 lx at the centre, and 5 lx at the corners. Mouse activity was analysed using a computer equipped with Activity Monitor v6.02 (Med Associates).

On the day mice were taken from the animal facility, they were allowed to acclimatise to the test chamber for 10 min with the overhead lighting off, then for 30 min with the lighting on. This acclimation process was repeated on the subsequent two days at an interval of 24 h. Previous studies revealed that daily repeated testing is possible up to three to four times a week without affecting the exploratory behavioural pattern^57,58^. On the day of the CSD or sham operation, the acclimation was carried out prior to the operation. At 24 h after CSD or sham operation, mice were intraperitoneally administered with either an active drug or vehicle (Fig. 1a). Subsequently, they were put into the testing chamber with the lighting off for 10 min, and their locomotive activity was recorded for the following 30 min with the lighting on. For all mice examined, behavioural testing was carried out between 10:00 and 18:00 in a quiet room.

### Assessment of mouse locomotion

The effects of drug administration on mouse locomotion in both compartments were evaluated using several parameters relevant to movement. The locomotion assessment was started at 10 min after administration with a drug or vehicle (Fig. 1b). In this experiment, when mice moved out of a 6.35 cm × 6.35 cm square box around them within 0.5 s, their movements were defined as ambulatory. Ambulatory distance (cm) was defined as the total distance travelled during the ambulatory movement status. Ambulatory time proportion was calculated by dividing ambulatory time by total time spent in a zone. Ambulatory average velocity (cm/s) was calculated as ambulatory distance divided by ambulatory time, which was defined as the time spent in the ambulatory movement status. In the present study, the ambulatory average velocity was used as an index to evaluate individual mouse motor function. We initially analysed behavioural data collected during the whole test period (30 min). In addition, we divided the test period into three 10-min-long segments to elucidate the temporal trajectories of locomotive parameters. Our preliminary experiments revealed that the standard deviation of the whole time spent in the light zone of untreated control mice was 120–150 s. With the type I error rate and power being 5% and 0.80, respectively, if we were to detect a 200-s difference, the sample size required was calculated as 7–10 subjects in each group.

### Statistical analyses

All numerical data are expressed as the mean ± SEM. Between-group comparisons of physiological parameters, CSD-related electrophysiological data and motility-relevant parameters were conducted with Kruskal–Wallis test, followed by Dunn’s multiple comparison test. The two-way repeated-measures ANOVA was used to evaluate the effects of CSD induction and pharmacological interventions on temporal trajectories of threshold temperature of heat pain, ambulatory time and distance. Post-hoc multiple comparisons were carried out using Bonferroni’s test. Mean differences were indicated with a 95% CI. Data were analysed using GraphPad Prism 7 software (GraphPad Software, San Diego, CA).

## Supplementary information

Supplementary file 1 (DOCX 16 kb)
