# Supplementary material for: Cortical spreading depolarisation-induced facial hyperalgesia, photophobia and hypomotility are ameliorated by sumatriptan and olcegepant
Source: Sci Rep. 2020 Jul 10;10:11408. doi: 10.1038/s41598-020-67948-w (PMC7351983; doi:10.1038/s41598-020-67948-w)
Supplement: Supplementary file 1 — (DOCX 16 kb) [file 41598_2020_67948_MOESM1_ESM.docx]

**Supplementary Table 1. Pre-operative ambulatory data among experimental groups.**

|  | Ambulatory time (s) | |  | Ambulatory distance (cm) | |
| --- | --- | --- | --- | --- | --- |
| Group | Light | Dark |  | Light | Dark |
| Sham-Veh | 47.5 ± 5.3 | 58.1 ± 5.6 |  | 1739.7 ± 215.0 | 2136.4 ± 187.0 |
| CSD-Veh | 49.8 ± 5.7 | 63.1 ± 5.7 |  | 1740.2 ± 191.3 | 2335.7 ± 187.3 |
| CSD-Suma | 53.5 ± 5.9 | 60.3 ± 6.7 |  | 1932.5 ± 217.8 | 2240.4 ± 200.9 |
| CSD-Olc0.25 | 55.1 ± 9.7 | 64.2 ± 5.4 |  | 1847.9 ± 315.9 | 2330.4 ± 180.9 |
| CSD-Olc1.0 | 48.4 ± 5.1 | 57.8 ± 3.8 |  | 1753.6 ± 196.3 | 2163.4 ± 143.4 |
